# *VEGF*, *FGF1*, *FGF2 *and *EGF *gene polymorphisms and psoriatic arthritis

**DOI:** 10.1186/1471-2474-8-1

**Published:** 2007-01-04

**Authors:** Christopher Butt, Sooyeol Lim, Celia Greenwood, Proton Rahman

**Affiliations:** 1 Discipline of Genetics, Faculty of Medicine, Memorial University of Newfoundland, St. John's, Newfoundland and Labrador, Canada; 2 Genetics and Genome Biology, Hospital for Sick Children, University of Toronto, Toronto, Ontario, Canada; 3 Division of Rheumatology, St. Clare's Mercy Hospital, Memorial University of Newfoundland, St. John's, Newfoundland and Labrador, Canada

## Abstract

**Background:**

Angiogenesis appears to be a first-order event in psoriatic arthritis (PsA). Among angiogenic factors, the cytokines vascular endothelial growth factor (VEGF), epidermal growth factor (EGF), and fibroblast growth factors 1 and 2 (FGF1 and FGF2) play a central role in the initiation of angiogenesis. Most of these cytokines have been shown to be upregulated in or associated with psoriasis, rheumatoid arthritis (RA) or ankylosing spondylitis (AS). As these diseases share common susceptibility associations with PsA, investigation of these angiogenic factors is warranted.

**Methods:**

Two hundred and fifty-eight patients with PsA and 154 ethnically matched controls were genotyped using a Sequenom chip-based MALDI-TOF mass spectrometry platform. Four SNPs in the *VEGF *gene, three SNPs in the *EGF *gene and one SNP each in *FGF1 *and *FGF2 *genes were evaluated. Statistical analysis was performed using Fisher's exact test, and the Cochrane-Armitage trend test. Associations with haplotypes were estimated by using weighted logistic models, where the individual haplotype estimates were obtained using Phase v2.1.

**Results:**

We have observed an increased frequency in the T allele of *VEGF *+936 (rs3025039) in control subjects when compared to our PsA patients [Fisher's exact p-value = 0.042; OR 0.653 (95% CI: 0.434, 0.982)]. Haplotyping of markers revealed no significant associations.

**Conclusion:**

The T allele of *VEGF *in +936 may act as a protective allele in the development of PsA. Further studies regarding the role of pro-angiogenic markers in PsA are warranted.

## Background

Psoriatic Arthritis (PsA) is an inflammatory form of arthritis usually seronegative for rheumatoid factor [1], which may affect as many as 30% of patients with psoriasis [2,3]. Although psoriasis and psoriatic arthritis (PsA) are interrelated disorders, PsA is a distinct entity with its own epidemiological clinical and genetic features. Furthermore, PsA demonstrates much greater heritability among first degree relatives (λ_1 _48) than psoriasis (λ_1 _5–10) [4].

Angiogenesis appears to be a first-order event in both psoriasis and PsA [5]. Abnormalities in the vascular morphology of the nail-folds of psoriasis patients without nail disease have been observed [6], as well as an increase in the number of synovial membrane blood vessels in PsA joint tissue [7]. Recently, the peroxisome proliferator activated receptor-γ (PPARγ) agonist pioglitazone, which inhibits angiogenesis, has shown efficacy in the treatment of PsA in a small open-label study [8]. Modest significance between a coding SNP of *PPARγ *and PsA patients has been observed [9], suggesting that angiogenesis may be an important area of investigation in PsA.

Among angiogenic factors, the cytokines vascular endothelial growth factor (*VEGF*), epidermal growth factor (*EGF*), and fibroblast growth factors 1 and 2 (*FGF1 *and *FGF2*) are powerful mitogens and play a central role in the initiation of angiogenesis. *VEGF *is the only mitogen that specifically acts on endothelial cells, and has been shown to stimulate the elongation, network formation, and branching of nonproliferating endothelial cells in culture that are deprived of oxygen and nutrients. As endothelial cells in tumors are routinely exposed to periodic or constant hypoxia, it has been proposed that *VEGF *contributes to the formation of blood vessels in tumorigenesis [10]. *FGF1 *has been shown to play a role in both angiogenesis and tumorigenesis [11], while *FGF2 *has been shown to play a crucial role in both skin and cartilage wound healing [12,13], and in combination with another pro-angiogenic factor synergistically induces vascular networks which remain stable for more than a year even after depletion of angiogenic factors [15]. *EGF *likewise is important in wound healing and plays a role in tumor growth and development [16].

High levels of the cytokine VEGF have been found in the synovial joints of both early and established rheumatoid Arthritis (RA), PsA [17], ankylosing spondylitis, [18], and psoriasis plaques [19]. The treatment of PsA with TNF-α inhibitors has been shown to significantly reduce levels of circulating VEGF and FGF1 [20].

Several *VEGF *polymorphisms have been associated with the development of psoriasis [21,22]. The 936 T allele (rs3025039) of the *VEGF *gene, and haplotypes at positions -2578 (rs699947), -1154 (rs1570360), -634 (rs2010963) and 936 have been associated with younger age of onset of RA in a Korean population [23]. Furthermore a haplotype of SNPs at positions -2578, -1154 and -634 has been associated with disease severity for ankylosing spondylitis in the same population [24].

Both *FGF1 *and *FGF2 *have been observed to be upregulated in the synovial tissue of human subjects with RA [25] and significantly worsened clinical symptoms in a rat adjuvant-induced model of arthritis [26], while at least one polymorphism of the *EGF *gene (rs4444903) has been shown to significantly affect *EGF *production [27]. At the current time, genetic variations in the *FGF1*, *FGF2 *or *EGF *genes have not been studied in either RA or the spondyloarthropathies.

Given the previously reported associations of *VEGF *in both RA and AS, and the role that *VEGF*, *FGF1*, *FGF2 *and *EGF *play in angiogenesis, we examined genetic variants of each of these genes in PsA subjects from Newfoundland.

## Methods

This study was approved by the local ethics committee of Memorial University of Newfoundland. Informed consent was obtained from all patients. All PsA probands were Caucasians from the Newfoundland population. PsA was diagnosed as inflammatory arthritis in patients with psoriasis, in the absence of any other etiology for inflammatory arthritis. Information was collected systematically and included age at onset of psoriasis, PsA and disease pattern. The ethnically matched, healthy control subjects were also obtained from the Newfoundland population, and were unrelated to the cases.

Whole blood samples were obtained from PsA probands and control subjects. DNA was extracted using the Promega Wizard Genomic DNA purification Kit. The detection of SNPs was performed by the analysis of primer extension products generated from previously amplified genomic DNA using a Sequenom chip-based MALDI-TOF mass spectrometry platform. In brief, PCR and extension reactions were designed using MassARRAY design software, and were carried out using 2.5 ng of template DNA. Unincorporated nucleotides in the PCR product were deactivated using shrimp alkaline phosphatase. The amplification of the SNP site was carried out using the MassExtend primer and involved the use of specific d/ddNTP termination mixes which were also determined using MassARRAY assay design software. The primer extension products were then cleaned and spotted onto a SpectroChip. The chips were scanned using a mass spectrometry workstation (Bruker) and the resulting spectra were analyzed and genotypes were determined using the Sequenom SpectroTYPER-RT software. We genotyped PsA probands and control subjects for the following polymorphisms: in the *VEGF *gene rs3025039, rs699947, rs1570360, and rs2010963; in the *FGF1 *gene rs34011; in the *FGF2 *gene rs1048201; and in the *EGF *gene rs4444903, rs11568943 and rs2237051.

For 2 × 2 contingency tables of allele frequencies, Fisher's exact tests were conducted to calculate the exact p-values, and odds ratios were also calculated. As supporting exploratory analyses, genotype frequencies were also examined: for each of the 2 × 3 contingency tables of genotype frequencies, two different statistical methods that require somewhat different modeling assumptions were used to generate p-values: one was Fisher's exact test, and the other was the Cochrane-Armitage trend test, which may have more power than Fisher's exact test if a trend exists across genotype categories under the additive genetic effect model.

Haplotype estimation was conducted in several stages using two software packages (Haploview [28] and Phase v2.1 [29,30]): Haploview was first run on the markers for each multi-marker gene to identify the linkage disequilibrium structure and check to ensure that the markers were appropriate for inclusion in the haplotype estimates. Once the markers for which haplotypes were to be constructed were identified for each gene, then Haploview was re-run to identify the likely haplotypes and determine their relative frequencies. In order to predict the haplotypes for each subject, the Phase software was run on the markers used for Haploview. Haplotyping was performed on the genes *EGF *and *VEGF *only, as there was only one genotyped marker in each of *FGF1 *and *FGF2*. Haplotype odds ratios for disease associations were calculated using the mixture logistic regression method proposed by Sham et al. (2004) [31], fitted by using WinBUGS 1.4 (Medical Research Council Biostatistics Unit, Cambridge) [32], which estimates Bayesian model parameters by using Markov chain Monte Carlo methods.

## Results

Two hundred and fifty-eight PsA probands and 154 ethnically matched controls were studied. All subjects were Caucasian of North European descent and considered to be native Newfoundlanders. The mean age of PsA patients at the time of study was 49.67 years (sd 10.95 years), and 48.5% of subjects were females. The mean age of onset of psoriasis in our cohort was 29.27 years (sd 14.16 years). Not all SNPs were successfully genotyped in every individual. The results of the genotyping experiments are given in Table 1.

Chi squared (χ^2^) tests for departure from the Hardy-Weinberg equilibrium were performed on each marker by Haploview The only marker that attained statistical significance in the control sample is rs4444903 in *EGF *gene (p < 0.01), and this marker was removed from further analysis; All the other markers satisfied H-W equilibrium.

The marker *VEGF *+936 (rs3025039) yielded a statistically significant result when Fisher's exact test was performed to compare allele frequencies between cases and controls (see Table 1). There was a higher proportion of T alleles among controls than cases (cases 11.6% vs. 16.8% in controls); Fisher's exact p-value = 0.042, OR for the T allele: 0.653 (95% CI: 0.434, 0.982). This finding is consistent with those of the statistical tests on the genotype frequencies (Fisher's exact test p = 0.082; Trend test p = 0.035).

The marker *VEGF *-634 (rs2010963) also appears to yield a statistically significant result when Fisher's exact test was performed on the genotype frequencies (p = 0.037). However, neither the Fisher's exact test on the allele table (p = 0.74) nor the trend test on the genotype table (p = 0.71) was statistically significant. The remaining two SNPs in the *VEGF *gene, *VEGF *-1154 (rs1570360) nor *VEGF *-2578 (rs699947) displayed no evidence of an association with PsA. For the *FGF1 *and *FGF2 *markers (rs34011 and rs1048201 respectively), there were no statistically significant findings in any of the tests (p > 0.5) that would suggest an association with PsA. For the two *EGF *SNPs, there was no evidence of association for either rs2237051 or rs11568943 in PsA.

The *VEGF *marker +936 (rs3025039) is visibly physically far from, and has low LD with, the other 3 markers in the gene. Thus, it was decided that rs3025039 should be excluded when estimating multi-marker haplotypes within this gene, (this decision is consistent with the *VEGF *haplotyping work of Seo et al [24]) and only rs699947, rs1570360, and rs2010963 were considered for haplotyping. There are 3 main haplotypes (AAG, CGC, and CGG) at the loci rs699947, rs1570360, and rs2010963, and haplotype frequency estimates are shown in Table 2. Haploview performed χ^2 ^tests comparing frequencies of haplotype CGC and CGG, respectively, in cases and controls. For CGC, the haplotype frequency was 0.325 in controls and 0.308 in cases, while the χ^2 ^test gave a p value of 0.612. For CGG the haplotype frequency was 0.138 in controls and 0.186 in cases, while the χ^2 ^test gave a p value of 0.077.

In order to calculate haplotype odds ratios (HOR), Phase v2.1 was used to make haplotype predictions for individuals. The haplotype frequencies generated by Phase and Haploview were compared and it was determined that the results from the two software packages were consistent with each other. A logistic regression model was then fitted to the case-control data to determine the odds ratio associated with each haplotype. Since haplotypes for some individuals cannot be precisely determined due to phase ambiguities, the mixture logistic regression method [31] was used to account for the probabilistic determination of haplotypes for individuals. This was implemented in WinBUGS 1.4. Note that this implementation does not yield p-values due to its Bayesian nature, but statistical significance can still be assessed by checking whether the 95% CI of HOR spans 1.0.

The results of the HOR analysis for the *EGF *and *VEGF *markers are given in Tables 3 and 4. Neither of the haplotypes for either gene was found to be strongly related to the disease status. It was decided that the marker rs4444903 of the *EGF *gene should be excluded from haplotyping as it was not in Hardy-Weinberg equilibrium in the control sample. Thus, only *EGF *markers rs11568943 and rs2237051 were considered for haplotyping. No evidence of a haplotype association with PsA for *EGF *markers (rs11568943 and rs2237051) was observed.

Of the 258 PsA probands, 164 had polyarticular disease (63.6%); 79 had oligoarticular disease (30.6%); 5 patients had isolated DIP variant (1.9%), 5 had isolated spondyloarthropathies (1.9%) and 3 had arthritis mutilans (1.1%).

Due to the low frequency of the isolated DIP, isolated spondylitis, and arthritis mutilans variant, we assessed differences in allele frequencies for our markers in between the oligoarticular and polyarticular subtypes of PsA, (data summarized in Table 5).

With respect to axial involvement, as noted above isolated spondylitis was relatively rare in our cohort. Most of the cases of spondylitis occurred in conjunction with either oligoarthritis or polyarthritis. Thus we stratified our PsA probands as having spondylitis or no spondylitis irrespective of peripheral involvement. We noted that 40 (15.5%) of our patients had a concomitant spondyloarthropathy and 201 patients (78.0%) had no axial involvement. For the remaining 17 patients there was insufficient information to properly assess for the presence of spondylitis (data summarized in Table 6). As noted in tables 5 and 6, no significant differences in minor allele frequencies were noted between variants of PsA and any of the SNPs examined in our study using Fisher's exact test.

## Discussion

The proposed physiological role of the cytokines coded for by the *VEGF*, *FGF1*, *FGF2 *and *EGF *genes, along with the increased levels of these angiogenic factors in arthritic synovium, make these worthy targets for evaluation of genetic association study.

We have observed an increased frequency in the T allele of *VEGF *+936 (rs3025039) in control subjects when compared to our PsA patients [Fisher's exact p-value = 0.042; OR 0.653 (95% CI: 0.434, 0.982)] indicating that this SNP may play a protective role against the development of PsA. This is in contrast to Han et al. [23] who observed an association of the T allele with the development of RA. It is worth noting that the pattern of increasing vascularity in PsA synovial tissue has been shown to be markedly distinct from that observed in RA: PsA patients were shown to have predominantly tortuous, bushy vessels while RA patients had mainly straight, branching vessels [33]. This potentially indicates differing mechanisms of angiogenesis, lending further evidence to the idea that there are indeed different pathogenic mechanisms in RA and PsA, which is not necessarily surprising.

Although the -1154 G→A (rs1570360) and -634 G→C (rs2010963) *VEGF *variants were not associated with rheumatoid arthritis in white population from Spain [34], interesting observations were found in assessing patients with primary systemic vasculitides from North-western Spain. With respect to this, biopsy-proven giant cell arteritis (GCA) patients, who had severe ischemic complications exhibited a significantly increased frequency of *VEGF *-634 G allele compared with GCA patients not affected by ischemic complications or with healthy controls. Interestingly, patients carrying the *VEGF *-634 C allele, associated with high production of *VEGF *had significantly reduced frequency of severe ischemic events in the setting of this large and middle-sized blood vessel vasculitis. In this regard, the carriage rate of the risk allele G showed statistically significant skewing comparing GCA patients with severe ischemic events with the remaining GCA patients (GG + GC compared with CC). These results suggest a potential implication of the *VEGF *gene -634 G-->C polymorphism in the development of severe ischemic manifestations of GCA. High *VEGF *levels may have a compensatory effect supporting neoangiogenesis mechanisms that may protect GCA patients from the development of severe ischemic complications such as irreversible visual loss [35]. In contrast, in Henoch-Schonlein Purpura (HSP), a small-sized blood vessel vasculitis involving skin, gut and kidney, the high *VEGF *producing -1154 G allele was increased in HSP patients with nephritis compared with healthy controls. Similarly, the high *VEGF *producing -634 C allele was also increased in patients with nephritis compared to controls. The -1154G/-634C haplotype was also associated with susceptibility to HSP nephritis. Moreover, a protective effect against nephritis in patients with HSP was observed for the -1154A/-634G *VEGF *promoter haplotype. These results also suggest a potential implication of the VEGF -1154 G-->A and -634 G-->C polymorphisms in the development of nephritis in patients with HSP [36]. It is also worth noting that in several instances the frequency of disease-associated and other alleles have been shown to be markedly different between Caucasian and Asian populations [35,36] including alleles associated with RA and other auto-immune conditions [37], therefore, our observed differences in the allele frequency of *VEGF *+936 (rs3025039) from other published reports is not surprising.

## Conclusion

Thus, the first investigation of genetic variations of the pro-angiogenic cytokines *VEGF*, *FGF1*, *FGF2*, and *EGF *in PsA has produced some interesting results. We observed that the T allele of *VEGF *in +936 may act as a protective allele in the development of PsA, in contrast to other reports which have observed a higher frequency of the allele in RA patients. The possibility does remain that an association exists for novel SNPs in these genes which may affect transcription levels or cause other functional changes, or within regulatory genes for *VEGF*, *FGF1*, *FGF2 *and *EGF*. It is also quite possible that variants in genes for other molecules which function in angiogenesis may play a role in PsA. Further studies regarding the role of pro-angiogenic markers in PsA would be beneficial to help elucidate pathogenic pathways in this disease.

## Abbreviations

PsA (Psoriatic Arthritis), PPARγ (peroxisome proliferator activated receptor-γ), SNP (single nucleotide polymorphism), VEGF (vascular endothelial growth factor) EGF (epidermal growth factor), FGF1 (fibroblast growth factors 1), FGF2 (fibroblast growth factors 2), RA (Rheumatoid Arthritis), AS (Ankylosing Spondylitis), HOR (Haplotype Odds-Ratio), GCA (Giant Cell Arteritis), HSP (Henoch-Schonlein purpura)

## Competing interests

The author(s) declare that they have no competing interests.

## Authors' contributions

CB carried out the molecular genetic studies, participated in study design, and drafted the initial manuscript. SL and CG performed the statistical analysis and contributed to the draft of the manuscript. PR conceived of the study, and participated in its design and coordination and helped to draft the manuscript. All authors read and approved the final manuscript.

## Pre-publication history

The pre-publication history for this paper can be accessed here:



## Figures and Tables

**Table 1:** Genotype frequencies of SNPs in VEGF, FGF1 FGF2 and EGF in PsA patients and controls

	Genotype	NF PsA patients	Controls
*VEGF*+936	CC	200/258 (77.5%)	100/146 (68.5%)
rs3025039	CT	56/258 (21.7%)	43/146 (29.5%)
	TT	2/258 (0.8%)	3/146 (2.0%)
Allele Freq (T)		11.6%	16.8%
*VEGF*-1154	GG	69/257 (26.8%)	35/147 (23.8%)
rs1570360	GA	121/257 (47.1%)	69/147 (46.9%)
	AA	67/257 (26.1%)	43/147 (29.3%)
Allele Freq (A)		49.6%	52.7%
*VEGF*-2578	AA	68/257 (26.5%)	45/150 (30.0%)
rs699947	AC	124/257 (48.2%)	70/150 (46.7%)
	CC	65/257 (25.3%)	35/150 (23.3%)
Allele Freq (C)		49.4%	46.7%
*VEGF*-634	GG	118/255 (46.3%)	64/127 (50.4%)
rs2010963	GC	118/255 (46.3%)	45/127 (35.4%)
	CC	19/255 (7.4%)	18/127 (14.2%)
Allele Freq (c)		30.6%	31.9%
*FGF1*	CC	110/242 (45.5%)	74/154 (48.0%)
rs34011	CT	108/242 (44.6%)	64/154 (41.6%)
	TT	24/242 (9.9%)	16/154 (10.4%)
Allele Freq (T)		32.2%	31.2%
*FGF2*	CC	167/254 (65.7%)	99/154 (64.3%)
rs1048201	CT	79/254 (31.1%)	47/154 (30.5%)
	TT	8/254 (3.2%)	8/154 (5.2%)
Allele Freq (T)		18.7%	20.5%
*EGF*	AA	209/248 (84.3%)	122/153 (79.7%)
rs4444903	GA	4/248 (1.6%)	1/153 (0.7%)
	GG	35/248 (14.1%)	30/153 (19.6%)
Allele Freq (G)		14.9%	19.9%
*EGF*	GG	78/256 (30.5%)	55/154 (35.7%)
rs2237051	GA	138/256 (53.9%)	72/154 (46.8%)
	AA	40/256 (15.6%)	27/154 (17.5%)
Allele Freq (A)		42.6%	40.9%
*EGF*	GG	233/243 (95.9%)	140/149 (94.0%)
rs11568943	GA	10/243 (4.1%)	9/149 (6.0%)
	AA	0	0
Allele Freq (A)		2.1%	3.0%

**Table 2:** Haplotype frequencies for VEGF rs699947, rs1570360, and rs2010963, estimated by using PHASE version 2.1.

Haplotype	Frequency in PsA cases (N = 516) No. (%)	Frequency in controls (N = 300) No. (%)
CGC	159.0 (30.8%)	97.6 (32.5%)
CGG	96.0 (18.6%)	41.4 (13.8%)
AAG	256.0 (49.6%)	156.9 (52.3%)
All other haplotypes	5 (0.97%)	4.1 (1.4%)

**Table 3:** Haplotype Odds Ratios (HOR) for PsA associated with EGF rs11568943 and rs2237051. The baseline haplotype for reference is GG.

EGF haplotype	HOR (95% CI)
GA	1.106 (0.824, 1.504)
AA	0.702 (0.264, 1.844)

**Table 4:** Haplotype Odds Ratio (HOR) analysis for VEGF rs699947, rs1570360, and rs2010963. The reference haplotype is AAG.

VEGF haplotype	HOR (95% CI)
CGC	0.946 (0.687, 1.294)
CGG	1.481 (0.932, 2.340)

**Table 5:** Minor allele frequencies in oligoarticular and polyarticular subsets of PsA. No significant differences were found between these two subgroups for any marker.

SNP	Minor allele frequency for Oligoarticular disease [N = 79; 30.6% of cases]	Minor allele frequency for Polyarticular disease [N = 164; 63.6% of cases]
VEGF +936 (T) rs3025039	0.12	0.11
VEGF -1154 (A) rs1570360	0.52	0.48
VEGF -2578 (C) rs699947	0.47	0.51
VEGF -634 (C) rs2010963	0.30	0.31
FGF1 (T) rs34011	0.32	0.33
FGF2 (T) rs1048201	0.21	0.18
EGF (G) rs4444903	0.16	0.15
EGF (A) rs2237051	0.46	0.42
EGF (A) rs11568943	0.01	0.03

**Table 6:** Minor allele frequencies in the spondyloarthritis subgroups of PsA. No significant differences were found between these two subgroups for any marker.

SNP	Minor allele frequency for cases with NO spondylitis [N = 201; 78.0% of cases]	Minor allele frequency for cases with Spondylitis present [N = 40; 15.5% of cases]
VEGF +936 (T) rs3025039	0.12	0.11
VEGF -1154 (A) rs1570360	0.48	0.53
VEGF -2578 (C) rs699947	0.5	0.45
VEGF -634 (C) rs2010963	0.31	0.29
FGF1 (T) rs34011	0.32	0.36
FGF2 (T) rs1048201	0.21	0.21
EGF (G) rs4444903	0.15	0.20
EGF (A) rs2237051	0.42	0.50
EGF (A) rs11568943	0.02	0.05
